# Emotional support for cancer patients: what do patients really want?

**DOI:** 10.1038/bjc.1996.529

**Published:** 1996-10

**Authors:** M. L. Slevin, S. E. Nichols, S. M. Downer, P. Wilson, T. A. Lister, S. Arnott, J. Maher, R. L. Souhami, J. S. Tobias, A. H. Goldstone, M. Cody

**Affiliations:** Department of Medical Oncology, St Bartholomew's Hospital, London, UK.

## Abstract

For many cancer patients and their families the experience of cancer is an intensely stressful one. Emotional support is important for most cancer patients during their illness and can be gained from different people and services. This study evaluates patients' attitudes to different sources of support and rates their satisfaction with sources already used. A total of 431 patients completed a questionnaire covering the use of different sources, including individuals, support groups and information sources. The questionnaire also incorporated validated measurements of anxiety, depression and locus of control. The results revealed that the three most important sources of emotional support were senior registrars (73%) and family (73%), followed by consultants (63%). Patients would prefer doctor- and nurse-led support groups to patient only-led groups (26% vs 12%). Pamphlets, such as the BACUP booklets, proved the most important of the informational sources sought (50%). A total of 86% of patients were satisfied or very satisfied with the emotional support received. Patients who expressed dissatisfaction with their emotional support were significantly more likely to be anxious and depressed (P < 0.001). Patients who used information sources were more likely to have a higher locus of control over the course of their disease. These results show how important the doctor's role is in the provision of emotional support.


British Journal of Cancer (1996) 74, 1275-1279
© 1996 Stockton Press All rights reserved 0007-0920/96 $12.00

### Emotional support for cancer patients: what do patients really want?

ML Slevin[1], SE Nichols[1], SM Downer[1], P Wilson[1], TA Lister[1], S Arnott[2], J Maher[3], RL Souhami[4], JS Tobias[5], AH Goldstone[6] and M Cody[1]

*Departments of [1]Medical Oncology and [2]Radiotherapy, St Bartholomew's Hospital, London; [3]Mount Vernon Centre for Cancer Treatment; [4]Department of Medical Oncology and [5]Meyerstein Institute of Oncology, Middlesex Hospital; [6]Department of Haematology, University College Hospital, London, UK.*

**Summary** For many cancer patients and their families the experience of cancer is an intensely stressful one. Emotional support is important for most cancer patients during their illness and can be gained from different people and services. This study evaluates patients' attitudes to different sources of support and rates their satisfaction with sources already used. A total of 431 patients completed a questionnaire covering the use of different sources, including individuals, support groups and information sources. The questionnaire also incorporated validated measurements of anxiety, depression and locus of control. The results revealed that the three most important sources of emotional support were senior registrars (73%) and family (73%), followed by consultants (63%). Patients would prefer doctor- and nurse-led support groups to patient only-led groups (26% *vs* 12%). Pamphlets, such as the BACUP booklets, proved the most important of the informational sources sought (50%). A total of 86% of patients were satisfied or very satisfied with the emotional support received. Patients who expressed dissatisfaction with their emotional support were significantly more likely to be anxious and depressed ($P<0.001$). Patients who used information sources were more likely to have a higher locus of control over the course of their disease. These results show how important the doctor's role is in the provision of emotional support.

**Keywords:** communication skills; emotional support; information provision

A diagnosis of cancer evokes a wide range of emotions, such as fear, anxiety, anger, depression, despair and helplessness. It can be a time of great emotional distress for patient and family. The patient is plunged from a state of apparently good health through a series of frightening transitions (Bloom, 1982): investigations and treatment with their potentially unpleasant side-effects, unanswered questions concerning recurrence, pain and death (Wortman and Dunkel-Schetter, 1979). With the uncertainties and loss of control comes a need for emotional support (Peters-Golden 1982; Winefield and Neuling, 1987; Broadhead and Kaplan, 1991). Patients need to understand what is happening to them and to be supported and reassured by others as to what will happen to them and whether their reactions are normal or otherwise (Wortman and Conway, 1985). Patients who receive strong and consistent emotional support are thought to adjust more successfully over time (Dunkel-Schetter, 1984).

Emotional support has been described as behaviour which assures the individual that he is loved and valued as a person regardless of achievement (Bloom, 1982; Cobb, 1976). It has also been defined in terms of physical presence, empathy, expressed concern, affection, others acceptance of patient's cancer, special understanding (Dakof and Taylor, 1990); love/concern, reassurance, encouragement (Dunkel-Schetter, 1984); and closeness with another person in whom the recipient can confide (Schaefer *et al.*, 1981). Different sources of support such as family, friends or doctors often provide different types of support (Rowland, 1990). For example, a doctor may offer information as a form of support, whereas family provide love and affection. In Dunkel-Schetter's 1984 study looking at the most helpful and unhelpful behaviours given to cancer patients, 'help' most often meant emotional support and was perceived as most supportive when given as a combination of information and direct help. Further studies have shown that information giving is an important predictor of satisfactory emotional support for patients (Blanchard *et al.*, 1990; Wortman and Dunkel-Schetter, 1979; Peck, 1972). By allowing patients to express their concerns, family and friends can acknowledge and help to manage their fears. Health professionals can discuss concerns and provide feedback about their experiences (Wortman and Dunkel-Schetter, 1979). This study aimed to find out from whom patients wished to receive emotional support and to assess the satisfaction with the various sources of support the patients had received at a number of NHS oncology units.

Correspondence: ML Slevin
Received 7 July 1995; revised 9 April 1996; accepted 7 May 1996

### Materials and methods

The questionnaire used was designed in-house and a general definition of emotional support was given as an introduction and a guide to the patients, which read as follows: 'Emotional support involves spending time with another person, listening and talking about problems and concerns in a way that is helpful and reassuring'. It is frequently acknowledged that a range of individuals contribute to a person's support network, including spouse, family and friends, health professionals and social support groups (Taylor *et al.*, 1986; Dunkel-Schetter, 1984; Wortman, 1984; Lewis and Bloom, 1978-1979). The aim of the study was to find out who patients would use as providers of emotional support if all sources of emotional support were freely available, and to rate their satisfaction with the support systems used already. The questionnaire also covered patients' views on the value of support groups and information sources as ways of gaining emotional support. Information sources included pamphlets and telephone counselling services. Given the increasing media coverage of cancer and its treatments, it was also felt to be worthwhile to include television and magazines.

Inclusion criteria were a diagnosis of cancer for at least 3 months, awareness of the diagnosis and ability to understand and read English. Patients were excluded if they had an inadequate understanding of their illness or if they were too unwell. Patients who were extremely anxious or depressed were also excluded, as it was felt it might be potentially distressing for them. This may have excluded some patients who were more likely to be referred to specialist services, such as to a psychiatrist. Patients were recruited from seven outpatient clinics, two medical oncology, three radiotherapy and two haematology clinics at four different hospitals, one of which was a district general hospital. This was to see if there were any major differences in the type of support sought and the satisfaction with and availability of sources of emotional support. There was a broad range of patients with different tumour types, who had been diagnosed for different lengths of time. The questionnaires were administered by oncology research nurses who spent an average of 20 min with each patient.

The questionnaire was divided into three sections:

Section 1: support from individuals. A list containing 17 individuals comprising a wide range of health care professionals as well as more peripheral carers, such as chaplain, psychologist and complementary therapist and non-medical people, such as family, friends and other patients.

Section 2: support groups, including self-help groups, groups led by doctors and nurses and psychologist- and psychiatrist-led groups, including a weekend seminar.

Section 3: emotional support from information and media sources, namely telephone, letter, pamphlets, magazines, television and books.

Patients were asked to rank each individual or group in each section by indicating whether they would definitely use, be likely to use, be unlikely to use or definitely not use. They were then asked if they had used that source. The questionnaire then divided the sources into five categories: doctors, nurses, support groups, information and non-medical people, and the patients were asked to rate whether they thought they were least important, not very important, very important or most important. Patients were then asked to make one single choice from that list, as the most important source of emotional support. The Hospital Anxiety and Depression (HAD) scale and the Cancer Locus of Control (LOC) scale were also included.

*Statistical methods*

To test for differences between proportions the chi-squared test was used, with Yates' correction where appropriate.

### Results

A total of 575 questionnaires were given out and 431 were returned, giving a response rate of 75%. The sample consisted of 270 females and 161 males. A total of 70% of patients were married or cohabiting (Table I).

The family and senior doctors were the two sources of emotional support patients would most like to use and these were rated as equally important (Figure 1). Altogether, 73% of patients would definitely use family, 73% would definitely use senior registrars and 63% would definitely use consultants as sources of emotional support.

A total of 52% of patients would also use friends for emotional support and 50% would use the ward sister. Although these were important sources of emotional support, they were secondary to a closer family network and more senior medical staff. Few patients said they would definitely use house officers (38%), and just 26% would use junior nurses. Overall, 43% of patients would definitely use general practitioners. Only 28% of patients said that they would definitely use other patients as a source of emotional support.

Satisfaction was rated by looking at those patients who had used a source and whether they indicated that they would definitely use one (Figure 2). There was clearly a high level of satisfaction for the three most important sources, family, senior registrar and consultant, around 80% appearing satisfied with the support received. Satisfaction was lower with the more junior staff; 59% had used house officers and, of those, about half would use them again. Satisfaction with GPs was higher than with house officers and other professionals, such as psychiatrists and psychologists; 63% of patients being satisfied with the emotional support from their GP. Less than half of patients who used ward nurses were satisfied with the emotional support received. Some 66% of patients had used other patients as a source of emotional support, and just 39% of those expressed satisfaction. This lack of enthusiasm to seek support from other patients is reflected in the results for the support groups (Figure 3). The numbers who would definitely use these were small in each group, but the doctor- and nurse-led groups were the ones the patients would most like to attend. Groups led by psychiatrists and psychologists were least likely to be wanted or used, although satisfaction was difficult to rate as the numbers of patients who had participated in these groups were very small.

The results from Section 3 clearly showed that pamphlets such as the BACUP booklets were the most important of the informational types of emotional support (Figure 4). In all, 50% of patients would definitely use pamphlets if they were available and 77% of those who had read them were satisfied. Regarding the media sources, 35% said they would definitely use television for information and 30% would read

**Table I** Demographic details

| | |
|---|---|
| Number | 431 |
| Median age (years) | 55 (Range 16–85 years) |
| M:F | 161:270 |
| Marital status | |
| Single | 13.5% |
| Married | 65.2% |
| Divorced | 4.6% |
| Separated | 1.1% |
| Widow/er | 10.0% |
| Cohabiting | 3.2% |
| Social class | |
| Professional | 4.6% |
| Intermediate | 17.2% |
| Skilled (non-manual) | 28.5% |
| Skilled (manual) | 19.0% |
| Partly skilled | 16.0% |
| Unskilled | 4.9% |

**Figure 1** Support from individuals – percentage who would definitely use.

**Figure 2** Support from individuals – percentage who would use again.

**Figure 3** Support from support groups – percentage who would definitely use.

magazines, but neither proved to be very popular, only 50% expressed satisfaction. More specialised books on cancer had a higher satisfaction rate. Patients were forced to make a choice between five different potential sources of emotional support, namely, family/friends, doctors, nurses, support groups and information sources (Figure 5). The results clearly illustrate that the two most important sources of emotional support are family and friends, and doctors, with the majority of patients reporting that doctors were more important than family and friends.

There was a significant difference in the age group of patients and the particular sources of emotional support chosen. Patients opting for health professionals, significantly consultants, senior registrars, GPs, community nurses and radiographers, were in the older age group, while younger patients were more likely to use family, friends and other patients. There was no association between types of support used and anxiety and depression, although patients who expressed dissatisfaction with the emotional support received had significantly higher anxiety and depression scores, as measured on the HAD scale ($P<0.001$). Patients who use

**Figure 4** Support from information sources – percentage who would definitely use.

**Figure 5** Emotional support – percentage who think most important overall.

information sources, particularly the pamphlets and cancer books, are more likely to have a higher locus of control over the course of their disease.

There were not too many differences across the seven different treatment centres in terms of sources of support sought by patients. Any differences occurring were mainly between radiotherapy and non-radiotherapy centres. Radiotherapy patients were more likely than oncology centre patients to want to seek support from radiographers, whereas oncology centre patients were more likely to want to seek support from ward nurses, SHOs and other patients. However, the finding for senior doctors was the same across the treatment centres. There were no differences in terms of satisfaction with the support received across the centres, e.g. district general *vs* teaching hospitals. Senior doctors and family were consistently rated as the supports with which patients were most highly satisfied.

### Discussion

Patients rated emotional support from senior doctors at least as highly as that from their family, and more important than any other source. Senior registrars, who see patients on a more regular basis than the consultant, were slightly more highly rated than their senior colleagues. It is the senior doctors who are perceived as having the most information and who make the decisions, thereby having most control over patients' well-being. Senior doctors also see the patients on a much more long-term basis, following them throughout the course of their illness, whereas junior doctors and nurses on rotation have a more transitory relationship. Despite the fact that senior doctors spend relatively little time with the patients, the high patient rating of senior doctors indicates that the quality of information given is valued more highly than the overall time spent. Blanchard *et al.* (1990) found that patients often overestimate the time they actually spend with a consultant. The satisfaction with the information provided in a very short time showed that more time does not necessarily result in better support. Her results found that information given by physicians was directly related to the patients' satisfaction with the emotional support received. Two other important studies carried out by Dunkel-Schetter (1984) and Dakof and Taylor (1990) showed that the most helpful action by doctors was the provision of information and the most unhelpful action was lack of information. Dunkel-Schetter's study found that physicians and health carers were mentioned about as frequently as family members as sources of greatest help and that competent medical care was viewed as unhelpful if not accompanied by informational or emotional support. Health carers were most effective when providing a combination of direct help and emotional support, as this was seen as a sign of caring and not just as resulting from obligation (Schaefer *et al.*, 1981). This suggests that one component of support cannot always work without another, and that there is considerable overlap between the types of support (Bloom, 1982; Wortman, 1984). The results presented here also show that ward sisters were seen as more important than junior nurses as providers of emotional support, again because of the seniority of their position. Patients, from their comments, generally find junior nurses helpful, kind and technically competent but too young or inexperienced to provide the emotional support they need. Some patients felt nurses had a problem with facing death and terminal illness. Several other studies have shown similar results regarding nurses, in that their actions paralleled that of intimate relationships; they were good at being pleasant and kind, but very few offered useful information (Dakof and Taylor, 1990; Peck, 1972), or were there to 'help the doctor' (Mitchell and Glicksman, 1977). The results show that GPs were rated higher than junior doctors, but less than senior hospital doctors, who have specialist knowledge and information. Psychiatrists and psychologists clearly provide great benefit to individual patients, but came lower in the rating. The study excluded patients thought to be particularly anxious and depressed, on the grounds that filling in questionnaires might be too distressing. This may have skewed the results and may explain why psychologists and psychiatrists were perceived as less important to this population. Many patients are also reluctant to be referred to mental health professionals because of the stigma or fear of having a mental illness label attached to them.

Pamphlets, such as the BACUP booklets (Slevin *et al.*, 1988), giving information on specific cancers and their treatments proved to be the most valuable and wanted of the information sources. As they provide specific information and are written by experts, they are more reliable than the generalised information provided by more media-oriented sources. Television programmes and magazines are accessible and widely used but satisfaction was low, and the information they present can sometimes be regarded as frightening and misleading.

The use of other patients as a source of emotional support relies on personal and individual experiences. While it often is enormously helpful for a newly diagnosed patient to talk to someone who has been through similar trauma, it can also be stressful (Brickman and Bulman, 1977). Patients have different attitudes to coping with cancer and it is not helpful for one patient who is trying to be positive to be a captive audience for another who is pessimistic. It has also been noted that patients who measure their own progress by using other patients who are doing well, may become distressed if they do not 'measure up' favourably (Sanders and Kardinal, 1977). It is likely that there are similar reasons explaining why so few patients attend support groups, although it has been suggested that patients join support groups when relationships with medical staff are unsatisfactory (Taylor *et al.*, 1986). In this sample of patients, those with a higher internal locus of control attended support groups and it may be that patients have to be very self-motivated to seek out groups.

Patients who scored highly on the anxiety and depression scale were much more likely to express dissatisfaction with the emotional support received. Patients who are lonely and depressed may well perceive few sources of emotional support for their deepest feelings and fears (Evans, 1975). Jamison *et al.* (1978) found that women who reported better emotional adjustment perceived their family, doctors and nurses as more supportive than women with lower adjustment.

### Conclusion

This survey demonstrates that patients regard their doctors as a very important source of emotional support. Senior doctors, in particular, were seen as very valuable. Wortman (1984) stated that provision of information itself can be seen as a type of support and this survey clearly shows that information is of paramount importance in providing emotional support. The delivery of information in a caring and sensitive manner may provide one of the most important sources of emotional support for cancer patients. It is vital, therefore, that doctors are trained in the necessary communication skills to elicit patients' needs for support and to respond appropriately to them. Doctors may complain that they do not have sufficient time to provide adequate information, but these results show that even a short amount of time spent with the patient is enormously beneficial. The study emphasises the pivotal role of senior medical staff in providing support for their patients. This, in turn, has very important implications for delivery of cancer care.

### References

BLANCHARD CG, LABRECQUE MS, RUCKDESCHEL JC AND BLANCHARD EB. (1990). Physician behaviours, patient perceptions and patient characteristics as predictors of satisfaction of hospitalised adult cancer patients. *Cancer*, **65**, 186–192.

BLOOM JR. (1982). Social support systems and cancer: a conceptual view. In *Psychosocial Aspects of Cancer*. Cohen J, Cullen JW and Martin LR (eds). Raven Press: New York.

BRICKMAN P AND BULMAN RJ. (1977). Pleasure and pain in social comparison. In *Social Comparison Processes*. Suls JM and Miller RL (eds). Hemisphere: Washington DC.

BROADHEAD WE and KAPLAN BH. (1991). Social support and the cancer patient. *Cancer*, **47**(suppl.), 794–799.

COBB S. (1976). Social support as a moderator of life stress. *Psychosom. Med.*, **38**, 300–314.

DAKOF GA AND TAYLOR SE. (1990). Victim's perception of social support: what is helpful from whom? *J. Personality Soc. Psychol.*, **58**, 80–89.

DUNKEL-SCHETTER C. (1984). Social support and cancer: findings based on patient interviews and their implications. *J. Soc. Issues*, **40**, 77–98.

EVANS J. (1975). Mastectomy: the patient's point of view. *Nursing Mirror*, **140**, 1.

JAMISON KR, WELLISCH DK AND PASNAU RO. (1978). Psychosocial aspects of mastectomy: 1. The woman's perspective. *Am. J. Psychiat.*, **134**, 432–436.

LEWIS FM AND BLOOM JR. (1978–79). Psychosocial adjustment to breast cancer: a review of the literature. *Int. J. Psychiat. Med.*, **9**, 1–17.

MITCHELL GW AND GLICKSMAN AS. (1977). Cancer patients: knowledge and attitudes. *Cancer*, **40**, 61–66.

PECK A. (1972). Emotional reactions to having cancer. *Am. J. Roentgenol. Radium Ther. Nucl. Med.*, **114**, 591–599.

PETERS-GOLDEN H. (1982). Breast cancer: varied perceptions of social support in the illness experience. *Soc. Sci. Med.*, **16**, 483–491.

ROWLAND JH. (1990). Interpersonal resources: social support. In *A Handbook of Psycho-oncology*. Holland JC and Rowland JH (eds). pp. 58–71. Oxford University Press.

SANDERS JB AND KARDINAL CG. (1977). Adaptive coping mechanisms in adult acute leukaemia patients in remission. *J. Am. Med. Assoc.*, **238**, 952–954.

SCHAEFER C, COYNE JC AND LAZARUS RS. (1981). The health related functions of social support. *J. Behav. Med.*, **4**, 381–406.

SLEVIN ML, TERRY Y, HALLETT N, JEFFRIES S, LAUNDER S, PLANT H, WAX H AND MCELWAIN T. (1988). BACUP - the first two years: evaluation of a national cancer information service. *Br. Med. J.*, **297**, 669–672.

TAYLOR SE, FALKE RL, SHOPTAW SJ AND LICHTMAN RR. (1986). Social support, support groups and the cancer patient. *J. Consulting Clin. Psychol.*, **54**, 608–615.

WINEFIELD HR AND NEULING SJ. (1987). Social support, counselling and cancer. *Br. J. Guidance Counselling*, **15**, 6–16.

WORTMAN CB. (1984). Social support in the cancer patient. Conceptual and methodological issues. *Cancer*, **53** (suppl.10), 2339–2362.

WORTMAN CB AND CONWAY TL. (1985). The role of social support in adaptation and recovery from physical illness. In *Social Support and Health*. Cohen S and Syme L (eds). Academic Press: New York.

WORTMAN CB AND DUNKEL-SCHETTER C. (1979). Interpersonal relationships and cancer: a theoretical analysis. *J. Soc. Issues*, **35**, 120–155.


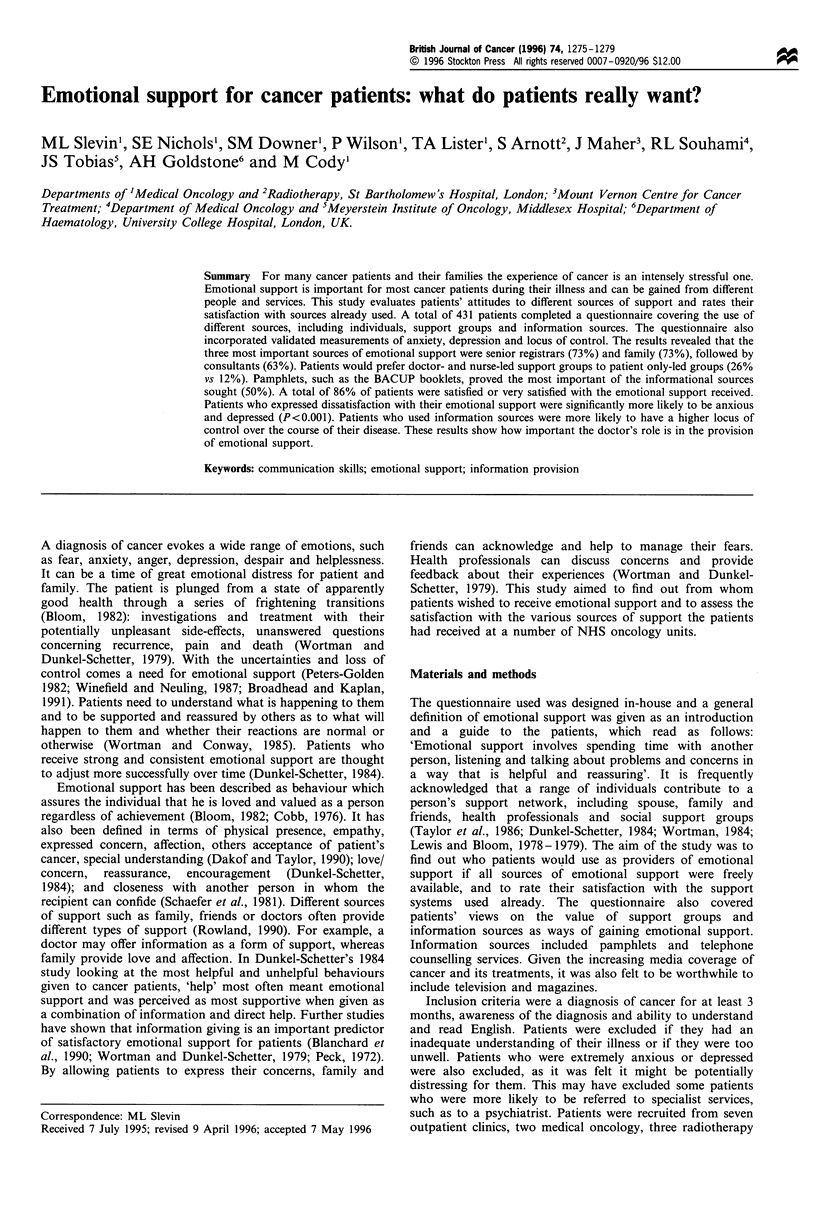

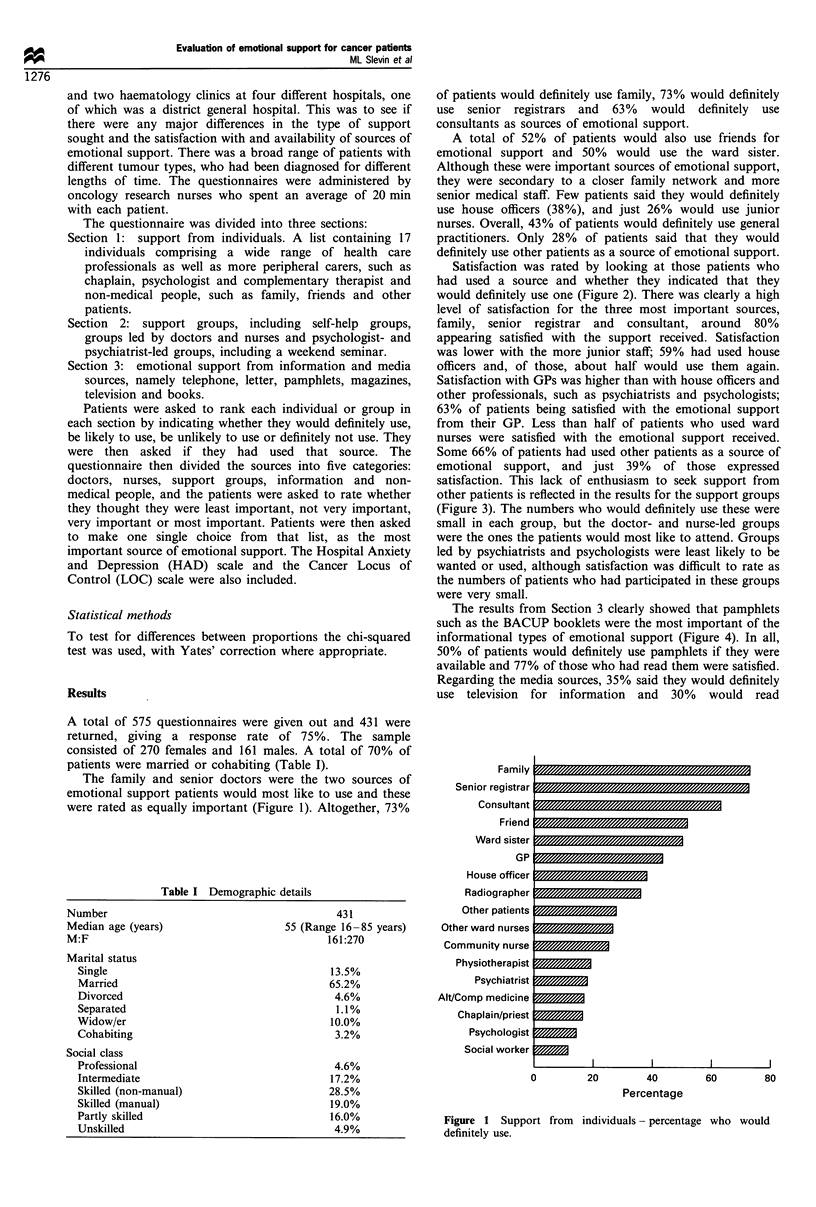

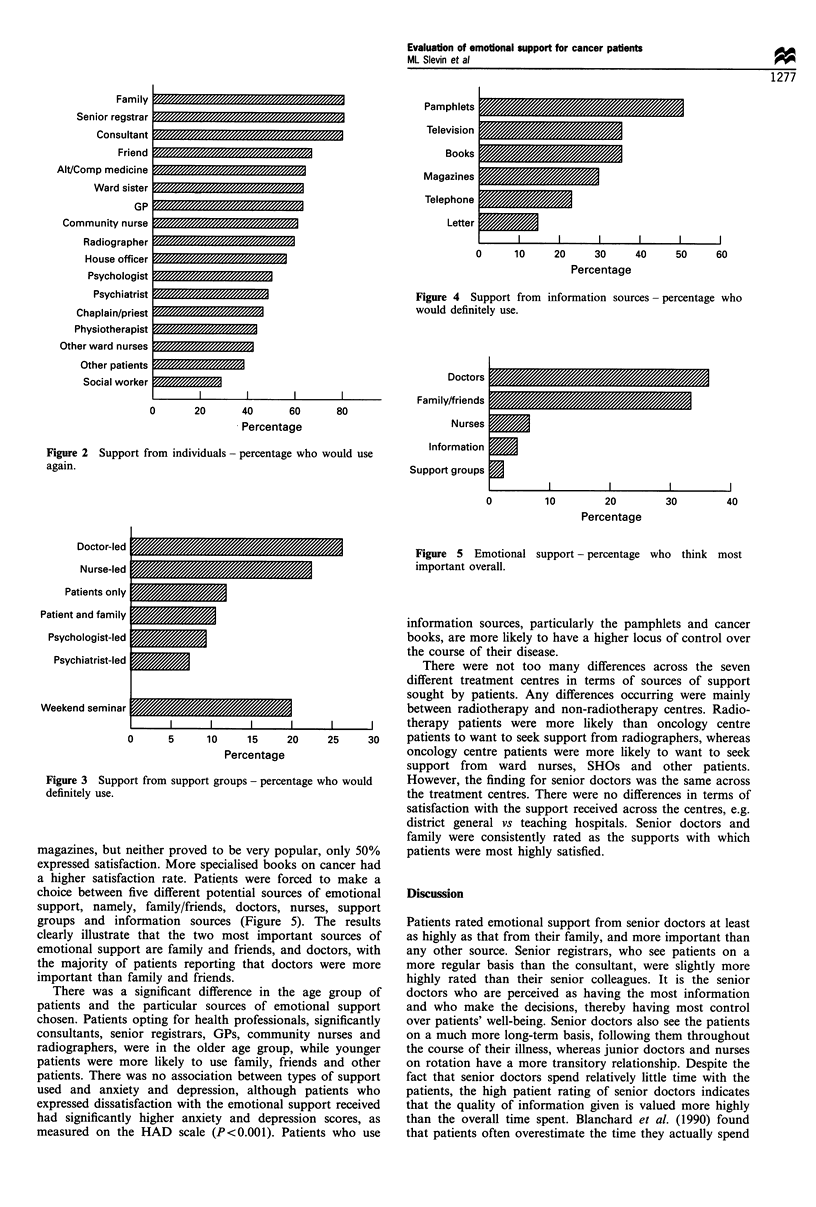

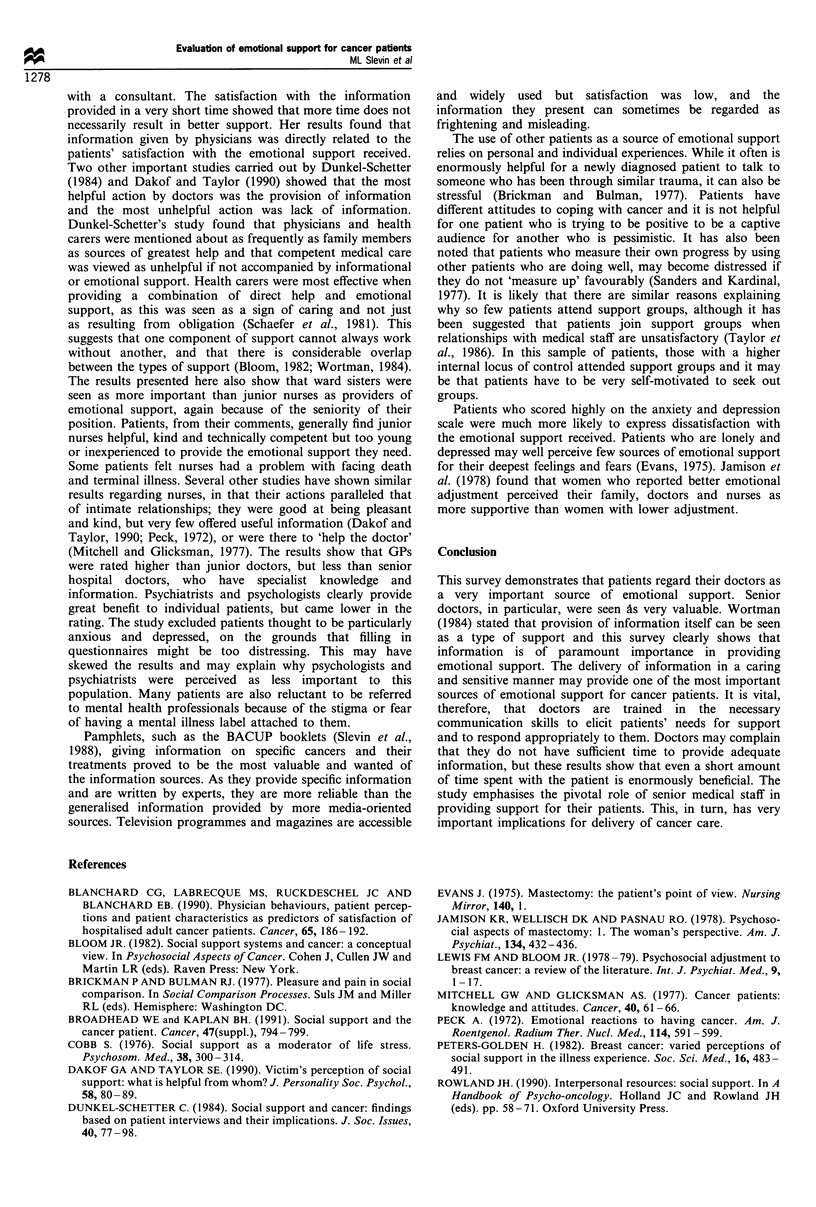

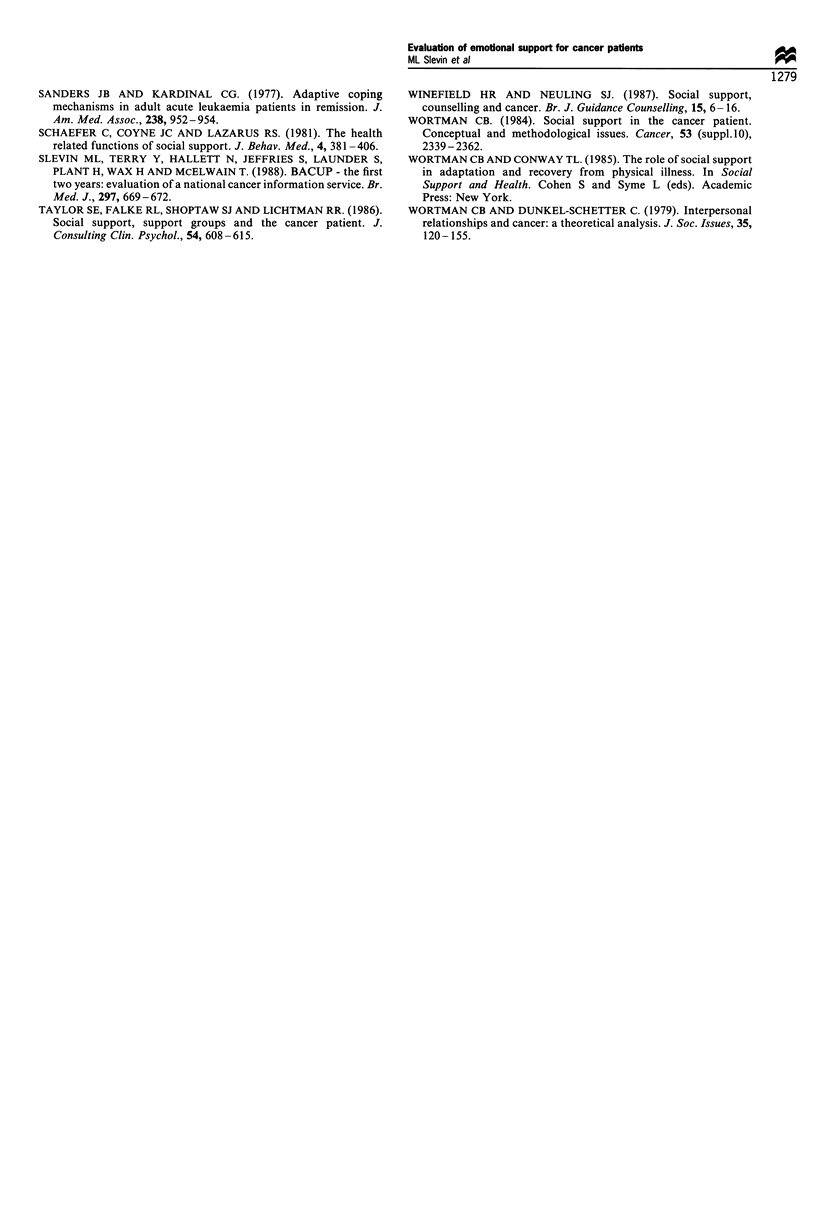


## References

[OCR_00643] Blanchard C. G., Labrecque M. S., Ruckdeschel J. C., Blanchard E. B. (1990). Physician behaviors, patient perceptions, and patient characteristics as predictors of satisfaction of hospitalized adult cancer patients.. Cancer.

[OCR_00659] Broadhead W. E., Kaplan B. H. (1991). Social support and the cancer patient. Implications for future research and clinical care.. Cancer.

[OCR_00665] Cobb S. (1976). Presidential Address-1976. Social support as a moderator of life stress.. Psychosom Med.

[OCR_00669] Dakof G. A., Taylor S. E. (1990). Victims' perceptions of social support: what is helpful from whom?. J Pers Soc Psychol.

[OCR_00682] Jamison K. R., Wellisch D. K., Pasnau R. O. (1978). Psychosocial aspects of mastectomy: I. the women's perspective.. Am J Psychiatry.

[OCR_00689] Lewis F. M., Bloom J. R. (1978). Psychosocial adjustment to breast cancer: a review of selected literature.. Int J Psychiatry Med.

[OCR_00694] Mitchell G. W., Glicksman A. S. (1977). Cancer patients: knowledge and attitudes.. Cancer.

[OCR_00696] Peck A. (1972). Emotional reactions to having cancer.. Am J Roentgenol Radium Ther Nucl Med.

[OCR_00700] Peters-Golden H. (1982). Breast cancer: varied perceptions of social support in the illness experience.. Soc Sci Med.

[OCR_00718] Sanders J. B., Kardinal C. G. (1977). Adaptive coping mechanisms in adult acute leukemia patients in remission.. JAMA.

[OCR_00723] Schaefer C., Coyne J. C., Lazarus R. S. (1981). The health-related functions of social support.. J Behav Med.

[OCR_00728] Slevin M. L., Terry Y., Hallett N., Jefferies S., Launder S., Plant R., Wax H., McElwain T. (1988). BACUP--the first two years: evaluation of a national cancer information service.. BMJ.

[OCR_00731] Taylor S. E., Falke R. L., Shoptaw S. J., Lichtman R. R. (1986). Social support, support groups, and the cancer patient.. J Consult Clin Psychol.

[OCR_00740] Wortman C. B. (1984). Social support and the cancer patient. Conceptual and methodologic issues.. Cancer.

